# The effect of the COVID-19 lockdown on malaria transmission in South Africa

**DOI:** 10.1186/s12936-023-04542-1

**Published:** 2023-03-24

**Authors:** Rajendra Maharaj, Abigail Ward, Bradley Didier, Ishen Seocharan, Nina Firas, Ryleen Balawanth, Dominic Lucero, Natashia Morris, Mbavhalelo Shandukani, Eric Raswiswi, Gillian Malatjie, Erik Mabunda, Devanand Moonasar

**Affiliations:** 1 Medical Research Council, Durban, South Africa; 2 Clinton Health Access Initiative, Boston, USA; 3 Clinton Health Access Initiative, Mbabane, Eswatini; 4 Clinton Health Access Initiative, Pretoria, South Africa; 5 Clinton Health Access Initiative, Gabarone, Botswana; 6 National Department of Health, Pretoria, South Africa; 7 KwaZulu-Natal Provincial Department of Health, Jozini, South Africa; 8 Mpumalanga Provincial Department of Health, Nelspruit, South Africa; 9 Limpopo Provincial Department of Health, Polokwane, South Africa; 10 World Health Organization, Pretoria, South Africa

**Keywords:** Malaria elimination, COVID-19, Imported malaria, Population movement

## Abstract

**Background:**

For a country such as South Africa which is targeting malaria elimination, mobile and migrant populations pose a substantial risk to importation of malaria parasites. It has been hypothesized that halting cross-border movement of mobile and migrant populations will decrease the importation of malaria, however this option is not a politically, operationally, and financially viable prospect. It has social impacts as well, since families live on either side of the border and preventing travel will challenge family ties. Due to the COVID-19 pandemic and closure of ports of entry (land and air) for non-essential travel into South Africa, a unique opportunity arose to test the hypothesis.

**Methodology:**

An interrupted time series analysis was done to assess whether the post-lockdown trends (April–December 2020) in monthly reported imported and local cases differed from the pre-lockdown trends (January 2015–March 2020). The analysis was conducted separately for KwaZulu-Natal, Mpumalanga, and Limpopo provinces.

**Results:**

On average, imported cases were lower in the post-intervention period in all three provinces, and local cases were lower in Mpumalanga and Limpopo, though no results were statistically significant.

**Conclusion:**

Since population movement continued after the travel restrictions were lifted, border screening with testing and treating should be considered for reducing parasite movement. Another option is reducing malaria cases at the source in neighbouring countries by implementing proven, effective vector and parasite control strategies and through a downstream effect reduce malaria entering South Africa.

## Background

Throughout history, humans have been plagued by diseases transferred from infected individuals to susceptible ones. Records indicate that pandemics were frequently vector-borne diseases and impacted on minor diseases elevating their importance in public health. Malaria, one of the oldest known epidemic diseases, remains a major problem of public health [1, 2]. It should be noted that malaria parasites are transported across borders in infected humans or vector mosquitoes.

Worldwide, malaria related morbidity and mortality has been on the decline since 2010 with 219 million cases and 405,000 deaths being reported in 2019 [3] as a result of increased resources being made available for malaria control. Nevertheless, malaria still causes widespread morbidity and mortality on the African continent especially amongst pregnant women and children under 5 years of age. Among the countries targeting malaria elimination in the next 5 years, progress has stalled and, in some cases, even reversed due to the non-implementation of effective malaria control interventions. The eight southernmost countries on the African continent are targeting malaria elimination by 2025 and 2030 through a collaboration called the Elimination 8 Initiative. Until malaria is eliminated, the region remains susceptible to upsurges such as those observed in 2017–2018, whether due to climatic conditions including increased rainfall, suboptimal interventions, or other causes such as increased importation into receptive areas. South Africa is one of the countries that aims to eliminate the disease by 2025, but the 2017 upsurge in cases may impede achievement of this target. The three endemic provinces in South Africa are in various stages of elimination [4, 5] with Limpopo province experiencing moderate transmission (> 1 local cases/1000 population), Mpumalanga low transmission (between 1 and 0.1 cases/1000 population) and KwaZulu-Natal very low transmission (< 0.1 cases/1000 population at risk) [6]. In 2000, KwaZulu-Natal reported the highest number of malaria cases due to insecticide and drug resistance. However through the implementation of effective control measures (new insecticide and combination therapy), KwaZulu-Natal is the lowest burden province. For the country to achieve elimination there are a number of challenges to overcome since residual malaria is hampering the elimination agenda especially in KwaZulu-Natal [7]. In a study by Raman et al*.* [8], along the border with South Africa and Mozambique, parasite isolates were genetically diverse and complex with limited genetic relatedness suggesting frequent and random mixing of parasites, consistent with the characteristics of imported infections from high transmission areas. Residual malaria and importation of parasites (through mobile and migrant populations) from neighbouring countries], are hampering the elimination agenda, especially in KwaZulu-Natal, putting receptive areas at constant risk of local outbreaks [8].

SARS-CoV-2, commonly referred to as COVID19, has wreaked havoc on health care systems and disease control programmes around the World. In response to the pandemic, different countries imposed various measures to contain and mitigate the effects of the virus. The first COVID-19 case imported into South Africa was on the 5^th^ March 2020 in a patient who had recently vacationed in Italy [9]. The South African government’s initial response to the COVID-19 pandemic was to declare a national state of disaster from March 2020. However, to stem the tide of imported cases into the country and to prevent the spread of the virus the country went into a state of lockdown which resulted in the cancellation of flights to and from South Africa and the closure of land border posts, to seal off the country from the rest of the world. To further prevent the spread of the disease within the country, inter-provincial travel was prohibited.

Whilst the national shutdown measures to help curb the spread of the virus resulted in widespread inconvenience, it created an unprecedented public health opportunity as well. It has long been hypothesized that interruption in the movement of infected persons (and, therefore, malaria parasites) across borders would impact the malaria elimination trajectory by reducing importation pressure on receptive areas [10]. The best way to test this hypothesis was to prevent cross-border movement of people, yet this could never be achieved in any conceivable manner as shutting the borders would be economically disastrous for South Africa and its neighbours. The measures implemented to stop the spread of the COVID-19 pandemic, which included border closures, were an opportunity to determine the effect on malaria cases in South Africa, especially during the ongoing COVID-19 pandemic. The key objective of the research inquiry was therefore to determine the effect that the lockdown (with border closures) had on malaria case importation from neighbouring countries into the malaria endemic provinces of South Africa, and the effect on local case transmission.

## Methods

### Data sources and data management

Since malaria is a medically notifiable condition, details on all cases diagnosed at health facilities are reported to the district health offices using standardised notification forms. Cases identified through active case detection are also routinely entered onto malaria notification forms which are submitted weekly to the provincial malaria control programmes where the data are entered onto a computerized Malaria Information System (MIS), as an interactive module in the District Health Information Software (DHIS2), which is a free and open-source health management data platform. The MIS allows for data entry at the individual patient level. Per the national malaria surveillance guidelines, all cases of malaria should be investigated within 48 h of diagnosis by malaria surveillance agents to determine the source of infection, and to classify them as local or imported. Imported cases are defined as those cases due to mosquito-borne transmission and acquired outside South Africa, including locally-imported cases, defined as cases imported from another part of the country, as indicated by the patient’s reported travel history. Local cases are defined as cases acquired due to mosquito-borne transmission and acquired within the country, either indigenous (contracted locally) or introduced (linked directly to a known imported case). A standard definition of imported malaria is used throughout the country for case investigation and classification. A standard travel history questionnaire is used to classify a case as local or imported. This is the same questionnaire that was administered before the pandemic, during the pandemic and currently, by the same case investigators. Thus, if the imported cases were over/underestimated, it would have been done so consistently thereby minimizing any major discrepancies.

To promptly identify cases and prevent the spread of disease in receptive areas, screenings at selected border entry points are conducted routinely. This measure was put into place to focus on asymptomatic carriage of malaria across borders into South Africa prior to the pandemic.

Raw monthly case data from January 2015 through December 2020 were extracted from the MIS for three endemic provinces (KwaZulu-Natal, Mpumalanga, and Limpopo).

### Data analysis

To align with border closures, which began on 26 March 2020 and ended on 29 December 2020, the “pre-lockdown” (pre) period was defined as January 2015 through March 2020 and the “lockdown” (post) period was defined as April to December 2020. The longitudinal trends pre and post border closures were compared through Poisson regression models fit separately to monthly imported and local case counts for each province using an interrupted time series analysis design [11]. Each model included one pair of Fournier (sine–cosine) terms, which introduces periodicity to adjust for seasonality [12]. Data analysis was conducted using Stata Statistical Software: Release 14 [13]. Within Stata, the glm command was used for regressions to ensure consistent standard errors, and a generalized chi-squared scale parameter was used to adjust standard errors since a population denominator was unknown for imported cases [12, 14]. The first outcome of interest was the difference in pre/post intervention trends, measured as IRR (incidence rate ratios) that approximate a relative risk, with a 95% confidence interval. Statistical significance was considered p < 0.05. The second outcome of interest was the difference between imported and local case trends within the same province. Because local cases are not independent of imported cases, these trends were compared descriptively.

### COVID-19 data

As outlined in Table 1, South Africa has five levels of covid-19 lockdown restrictions that were designed to restrict the spread of the disease [15, 16]. The covid-19 data used in this study was made available by the National Department of Health, South Africa. The main focus is on lockdown alert level 5 when international and inter-provincial travel was not permitted and people confined to their homes.

**Table 1:** Understanding the alert levels during the pandemic in South Africa

Alert level	Objective	Relevant restrictions
5	Drastic measures to contain the spread of the virus and save lives especially restrictions on travel	Every person is confined to their residence, Movement between provinces, metropolitan and districts are prohibited All land and air borders closed during the period of lockdown
4	Extreme precautions to limit community transmission and outbreaks, while allowing some activities to resume	Borders remain closed to international travel Travel between provinces permitted
3	Restrictions on many activities, including at workplaces and socially, to address a high risk of transmission	Limited domestic air travel
2	Physical distancing and restrictions on leisure and social activities to prevent a resurgence of the virus	Land borders remain closed, except for certain border posts domestic flights permitted
1	Most normal activities can resume, with precautions and health guidelines adhered to at all times	Restrictions on international travel

## Results

**Fig. 1:**
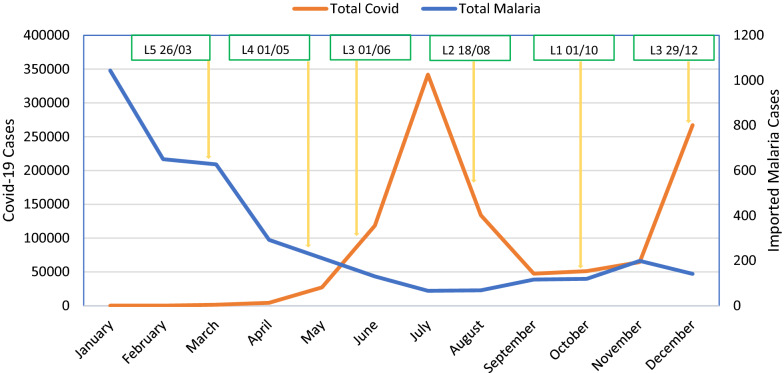
Malaria and COVID-19 cases for 2020 at the different lockdown levels (Level 1 (L1)–Level 5 (L5))

With the arrival of SARS-CoV-2 into South Africa, the country went into hard lockdown resulting in borders closures effectively halting the migration of people. The number of migrants was hypothesized to decrease during the lockdown and there was a corresponding change in the number of imported cases into the country (Fig. 1). At the peak of the first COVID-19 wave, the mitigation measures were at their most stringent and imported malaria cases were at their lowest.

### Differences in pre/post interruption trends

**Fig. 2:**
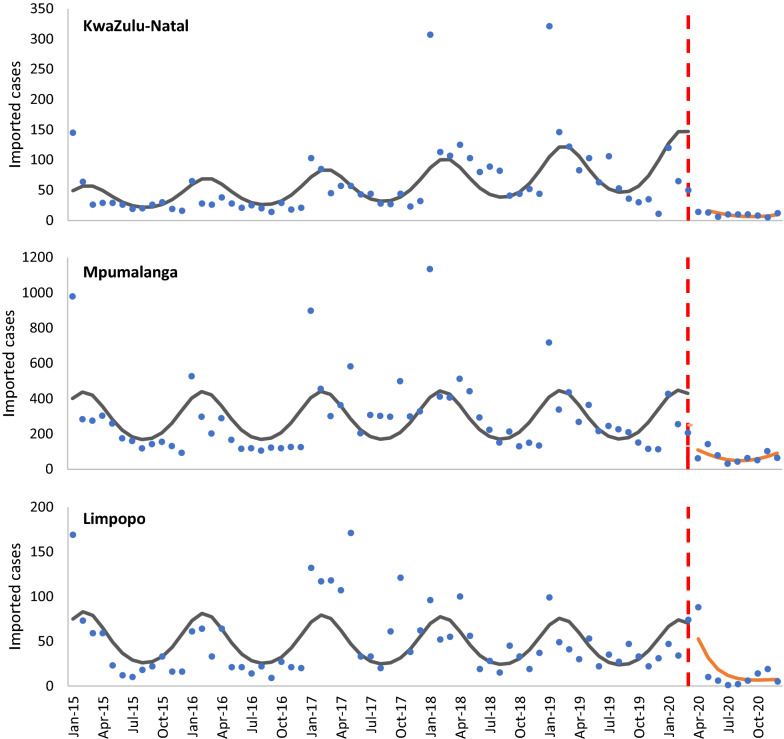
Imported malaria cases into the three endemic provinces (2015–2020)

On average, fewer imported cases per month were reported in the period after lockdown in all three provinces (Fig. 2); however, none of these differences in trend was statistically significant (KwaZulu-Natal IRR = 0.98, 95% CI [0.66–1.45]; Mpumalanga IRR = 0.99 [0.76–1.29]; Limpopo IRR = 0.79 [0.58–1.08]).

Table 2 shows the results of the regressions performed at province level for imported and local monthly reported cases.

**Table 2:** Regression results for local and imported cases (IRR and 95% confidence interval)

	Imported cases			Local cases		
	KwaZulu-Natal	Mpumalanga	Limpopo	KwaZulu-Natal	Mpumalanga	Limpopo
Trend pre interruption	1.02 [1.01–1.03]	1 [0.99–1.01]	1 [0.99–1.01]	1 [0.99–1.01]	1.01 [0.99–1.03]	1 [0.98–1.02]
Interruption (March to April 2020)	0.13 [0.01–1.15]	0.3 [0.07–1.37]	1.14 [0.32–4.11]	0.33 [0.04–2.74]	0.35 [0–31.06]	1.52 [0.18–13.17]
Difference in trends pre/post interruption	0.98 [0.66–1.45]	0.99 [0.76–1.29]	0.79 [0.58–1.08]	1.07 [0.75–1.52]	0.87 [0.4–1.89]	0.78 [0.46–1.34]
Trend post interruption	0.99 [0.67–1.47]	0.99 [0.76–1.29]	0.79 [0.58–1.08]	1.06 [0.75–1.52]	0.88 [0.41–1.91]	0.78 [0.46–1.34]

No results were statistically significant using p = 0.05 as a threshold

**Fig. 3:**
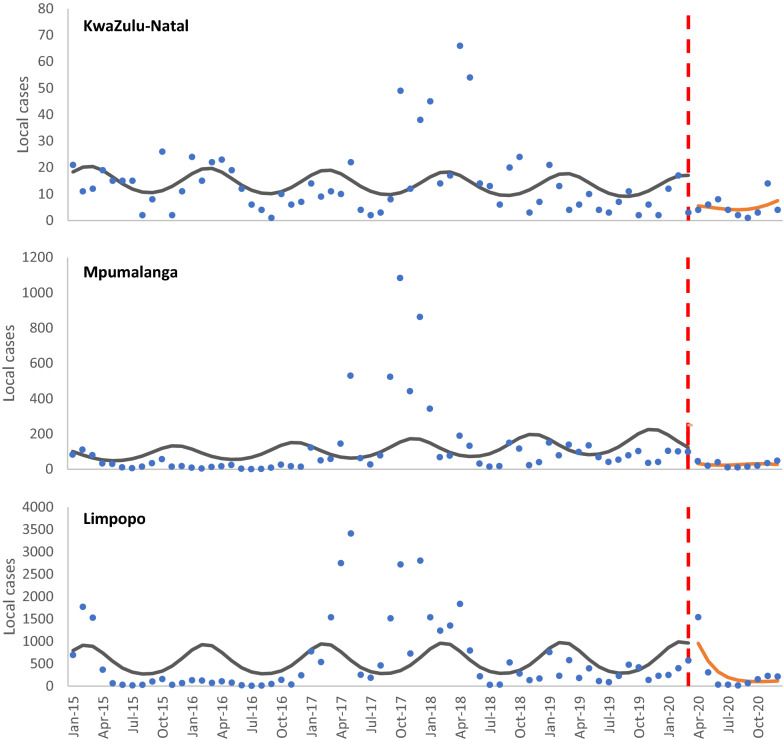
Local malaria cases in the three endemic provinces (2015–2020)

Figure 3 shows the local case data pre/post interruption with fitted trends. Fewer local cases per month were also reported on average in Mpumalanga and Limpopo in the post-interruption period (Mpumalanga IRR = 0.87 [0.4–1.89]; Limpopo IRR = 0.78 [0.46–1.34]). The local case trend in KwaZulu-Natal was slightly higher than pre-interruption (IRR = 1.07 [0.75–1.52], though none of the differences in local case trends pre/post intervention was statistically significant.

### Differences between imported and local trends within provinces

In KwaZulu-Natal, imported and local case trends changed (albeit slightly) in different directions. In Mpumalanga and Limpopo, declines were slightly larger in local cases than imported cases on average. At the time of interruption (between March and April 2020) reported imported and local cases fell in KwaZulu-Natal and Mpumalanga. Imported and local cases increased briefly between these months in Limpopo before falling.

## Discussion

The purpose of the national lock-down was to prepare and respond to the pandemic. As part of the COVID-19 preparedness and response plan, monitoring and surveillance systems were put in place to contain the spread of the virus. Malaria control measures and malaria control programme functioning was not impacted upon. Malaria case reporting followed the same seasonal trend since the lockdown was implemented mostly in the winter months of the transmission cycle. Moreover, the border districts in South Africa did not experience high numbers of COVID-19 cases as was seen in the peri urban and urban areas. Although the borders were closed, people were still entering the country illegally at informal border crossing sites.

In the endemic provinces the classification of these cases is not so easy and genetic studies is not feasible on every malaria infected individual. Foci of cases are investigated and in South Africa these foci usually stem from imported cases. During reactive case detection, communities are actively tested for malaria and the cases are classified according to guidelines set by the national Department of Health. Travel history questionnaires were developed by the national Department of Health in consultation with WHO and other partners, for use in identifying imported cases. The usefulness of this tool is negated by individuals who do not divulge their travel history but refusal to provide travel history occurred prior to the pandemic and during the pandemic so it was not biasing the data or skewing the data at any given point. Travel history-based classification indicates that hundreds of infected people cross into South Africa from high transmission neighbouring countries. Closure of international borders from 27 March 2020 did result in fewer reported imported cases on average, comparing pre/post-interruption trends, though none of the results are statistically significant.

The fact that there were any imported cases detected in the country showed that the porous borders could be enabling infected people to enter the country at informal border crossing points. Testing should be conducted on all persons entering the country, but the current diagnostic tests require individuals to wait 15–20 min, which would clog ports of entry and deter voluntary testing. Furthermore, RDT’s are not cost-effective when used for border screening and may have limited value due to possible resource limitations in such a setting. Newer diagnostic tests with a quick turn-around time are required, preferably one that is not invasive and is cost-efficient. Informal border crossings should be identified, and test and treat facilities set up in these areas to identify infected individuals coming into South Africa. Mobile clinics funded by the Department of Health, should also be located at these informal entry points so that health care can be provided as required. It is hypothesized that people crossing via the official border points would be more affluent and able to afford health care. It is likely that in this population the number of infected individuals would be low [17]. It is in these situations that the multi-country initiatives such as MoSaSwa (a collaboration between Mozambique, South Africa and Eswatini) will be useful to decrease the prevalence and incidence of malaria amongst those populations moving on both sides of the border [5, 18].

International travel has increased markedly since the last influenza pandemic in 1918 [19]. The number of people traveling for business or leisure numbers in the hundreds of millions of which about 43% of those travelling was by air [19]. This increase in international travel has heightened the risk for the global spread of infectious diseases as was evident by the rapid spread of COVID-19 from Wuhan province in China to Europe and the United States. In order to curtail and prevent the spread of disease, many countries have instituted border disease control measures as a response to a fast-spreading disease. Screening at border crossings, quarantine and isolation were some of the measures that were implemented during the SARS (severe acute respiratory syndrome) pandemic of 2003 and the influenza pandemic of 2009 [20]. In Africa especially, rapid and accessible long-distance road transportation facilitates the geographic spread of diseases, even those such as influenza, that have a short incubation period [20].

In a similar vein, malaria parasites can travel long distances and cause infections where there is a naïve population. Parasites travel in asymptomatic humans or in infected mosquitoes and the parasites travel ubiquitously via air and ground transportation in a similar fashion to viral diseases. Movement of malaria across international borders poses a major challenge in achieving malaria elimination. Wangdi [21] found that in border areas, malaria prevalence is often higher than in other parts of the country since access to health services is limited and delayed treatment-seeking behaviour results in unnecessary deaths or hospitalisation. Furthermore, there are difficulties in deploying prevention programs to marginalised communities, and constant movement of people across porous national boundaries makes it difficult to test, treat and track infected individuals. Key to addressing the challenges posed at borders is the strengthening of surveillance activities for rapid identification of any importation or reintroduction of malaria [21]. According to Miller et al. [22] the COVID-19 pandemic places an extra burden on health systems globally.

Resources for malaria often face challenges in the face of more novel disease threats, which can result in a resurgence of malaria. This was the case during the outbreak of Ebola in West Africa where resources targeted for malaria were reprogrammed into the efforts to control the new threat. The result of this was an unprecedented surge in malaria cases. During the lockdown in South Africa, the number of malaria control staff were reduced resulting in malaria control and prevention activities being minimal. Staff were re-deployed to assist with COVID-19 activities.

Regardless of the magnitude or the impact that COVID-19 has had on malaria transmission in the country, the easing of restrictions, particularly the opening of international borders, poses a major challenge in preventing cross-border malaria transmission. The WHO [3] had predicted huge increases in malaria cases and deaths if adequate, sustainable resources are not put toward implementing malaria elimination tools. Testing of individuals at border posts should be stepped up since it has been found that asymptomatic carriage of the disease can drive low level transmission [8].

Given the reliance of this analysis on passive case-based surveillance data, it does have some limitations. Due to the nature of the routine health system, it is possible that during COVID-19 there was reduced care seeking for febrile illness which led to lower detection rates of incident malaria. The border closures and focus on COVID-19 could also have reduced suspicion of malaria amongst healthcare workers, despite messaging to remain vigilant. During the COVID-19 lockdown it was possible that patients were reluctant to provide a true account of travel history due to official border closures and illegality of cross-border movement at that time. These infected individuals came across informal border crossing points and closure of the borders did not totally prevent movement of people into South Africa. During the pandemic, individuals were classified as imported cases mainly at clinics when they sought treatment for febrile illnesses. Additionally, the seasonal fitted curves are approximations of reality, as is clear by numerous outliers in the imported and local case trends. These outliers were retained for this analysis because they do not coincide in the timeline, indicating they are not due to anomalous situations such as long-term climate cycles or other major disturbances, but regular noise in the longitudinal data trend.

## Conclusion

KwaZulu-Natal, Mpumalanga, and Limpopo all experienced slight, non-significant reductions in the average imported cases reported after the March 2020 travel restrictions due to COVID-19. Mpumalanga and Limpopo also experienced non-significant declines in average local cases reported in the post-intervention period. Whilst this is not a viable long-term solution as trade and tourism would be severely affected, reduction in malaria at the sources of infection and border testing and treatment may be options for reducing malaria importation and subsequent onward local transmission into South Africa. The practicalities and costs for taking such measures needs to be gauged. Alternatively testing and treatment of malaria prior to entering lower transmission countries may be another option to pursue, i.e. reducing malaria at its source. This option is that is currently being explored through a collaboration between the South African and Mozambiquan Ministeries of Health in an attempt to reach zero cases in border districts of the two countries.

## Data Availability

Data used in this study is available from the principal author and was obtained from the Department of Health in South Africa.
